# Suppression of RANKL-Induced Osteoclastogenesis by the Metabolites from the Marine Fungus *Aspergillus flocculosus* Isolated from a Sponge *Stylissa* sp.

**DOI:** 10.3390/md16010014

**Published:** 2018-01-05

**Authors:** Hee Jae Shin, Byeoung-Kyu Choi, Phan Thi Hoai Trinh, Hwa-Sun Lee, Jong Soon Kang, Tran Thi Thanh Van, Hyi-Seung Lee, Jong Seok Lee, Yeon-Ju Lee, Jihoon Lee

**Affiliations:** 1 Marine Natural Products Chemistry Laboratory, Korea Institute of Ocean Science and Technology, 385, Haeyang-ro, Yeongdo-gu, Busan Metropolitan City 49111, Korea; choibk4404@kiost.ac (B.-K.C.); hwasunlee@kiost.ac (H.-S.L.); hslee@kiost.ac (H.-S.L.); jslee@kiost.ac (J.S.L.); yjlee@kiost.ac (Y.-J.L.); jihoonlee@kiost.ac (J.L.); 2 Department of Marine Biotechnology, University of Science and Technology, 217 Gajungro Yuseong-gu, Daejeon 34113, Korea; 3 Nhatrang Institute of Technology Research and Application, Vietnam Academy of Science and Technology, 02 Hung Vuong, Nha Trang 650000, Vietnam; phanhoaitrinh84@gmail.com (P.T.H.T.); tranthanhvan@nitra.vast.vn (T.T.T.V.); 4 Graduate University of Science and Technology, Vietnam Academy of Science and Technology, 18 Hoang Quoc Viet, Cau Giay, Ha Noi 100000, Vietnam; 5 Bio-Evaluation Center, Korea Research Institute of Biotechnology, 30 Yeongudanjiro, Cheongju 28116, Korea; kanjon@kribb.re.kr

**Keywords:** mactanamide, osteoclastogenesis, marine fungus, *Aspergillus flocculosus*, TRAP assay

## Abstract

A new α-pyrone merosesquiterpenoid possessing an angular tetracyclic carbon skeleton, ochraceopone F (**1**), and four known secondary metabolites, aspertetranone D (**2**), cycloechinulin (**3**), wasabidienone E (**4**), and mactanamide (**5**), were isolated from the marine fungus *Aspergillus flocculosus* derived from a sponge *Stylissa* sp. collected in Vietnam. The structures of Compounds **1**–**5** were elucidated by analysis of 1D and 2D NMR spectra and MS data. All the isolated compounds were evaluated for anti-proliferation activity and their suppression effects on receptor activator of nuclear factor κB ligand (RANKL)-induced osteoclast differentiation using tartate-resisant acid phosphatase (TRAP). Compounds **1**–**5** had no anti-proliferative effect on human cancer cell lines up to 30 μg/mL. Among these compounds, aspertetranone D (**2**) and wasabidienone E (**4**) exhibited weak osteoclast differentiation inhibitory activity at 10 μg/mL. However, mactanamide (**5**) showed a potent suppression effect of osteoclast differentiation without any evidence of cytotoxicity.

## 1. Introduction

Bone remodeling is the regulation to maintain the quality and mass of bone by undergoing a resorption and formation cycle repetitively [1,2]. The cells responsible for the skeleton resorption are the osteoclasts. Osteoclasts are multinucleated cells generated from their mononuclear precursor cells derived from the monocyte/macrophage lineage [3]. Osteoclasts are induced by two putative promoters, macrophage colony stimulating factor (M-CSF) and receptor activator of nuclear factor-κB ligand (RANKL). However, the excessive formation of osteoclasts causes abnormal bone remodeling, caused by the increase bone destruction. Most adult skeletal diseases such as osteoporosis, rheumatoid arthritis, and periodontal disease are associated with a slight imbalance between bone formation and bone resorption [4,5,6]. Thus, efforts have been devoted to the discovery of substances that inhibit RANKL-induced osteoclast differentiation for suppressing osteoclastogenesis [7,8].

**Figure 1:**
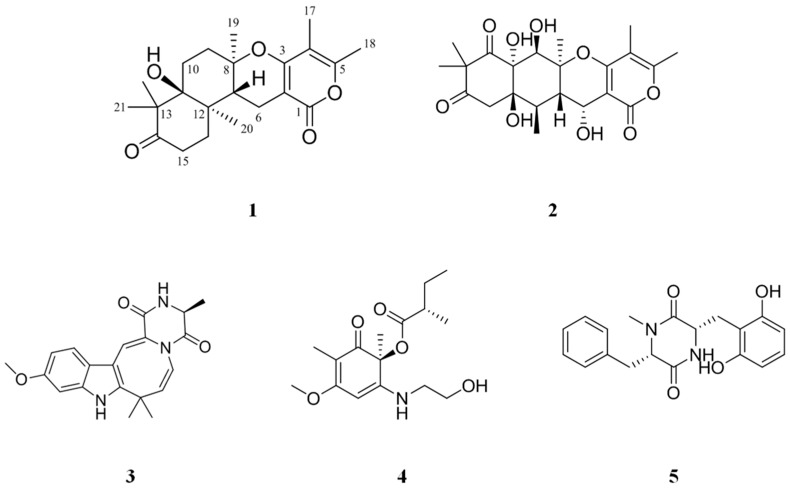
Structures of Compounds **1**–**5** isolated from *Aspergillus flocculosus.*

The majority of novel secondary metabolites from the ocean have structural diversities and potent bioactivities [9]. Many marine organisms, especially sponges, have been attracting significant attention from natural product chemists and biologists due to the potential of marine natural products as drugs during recent years [10]. It is known that some marine secondary metabolites from sponges may function as a chemical defense for associated microorganisms [11]. Moreover, natural products from microbial symbionts may also protect the sponge from environmental threats such as overgrowth, UV irradiation, and attack by feeder [9]. Associations between sponge and microorganisms gave rise to the idea that metabolites may be from their symbionts [12,13]. The structures of various natural products isolated from microorganisms closely resemble those identified in host animals [14,15]. As part of our continuing efforts to discover bioactive natural products from sponge-derived microorganisms, various marine sponges were collected and investigated for the isolation and identification of microorganisms. During studies on the diversity of fungi, a marine fungal strain 01NT-1.1.5, *Aspergillus flocculosus* derived from a sponge *Stylissa* sp., was isolated through experimental tools for the isolation of microorganisms. *Aspergillus* spp. and *Penicillium* spp. are well-known as the most chemically examined fungal genera [16,17]. Moreover, hundreds of structurally unique metabolites have been reported with interesting bioactivities as therapeutic candidates [18]. The fungus *A. flocculosus* 01NT.1.1.5 was originally isolated from the sponge *Stylissa* sp. collected at Nha Trang Bay, Vietnam, in February 2016. In preliminary screening, this strain showed good bioactivities such as antimicrobial and antifungal properties. The crude extract of the strain was purified further by a reversed-phase HPLC to yield one new compound, ochraceopone F (**1**), and four known compounds (Figures S8–S19), aspertetranone D (**2**) [19], cycloechinulin (**3**) [20], wasabidienone E (**4**) [21], and mactanamide (**5**) [22] (Figure 1). The structures of the known compounds were identified by 1D and 2D NMR analysis and comparison with literature data. All compounds were tested for anti-proliferative activity on human cancer cell lines and RANKL-induced osteoclast differentiation inhibitory effect using a TRAP assay.

## 2. Results and Discussion

**Figure 2:**
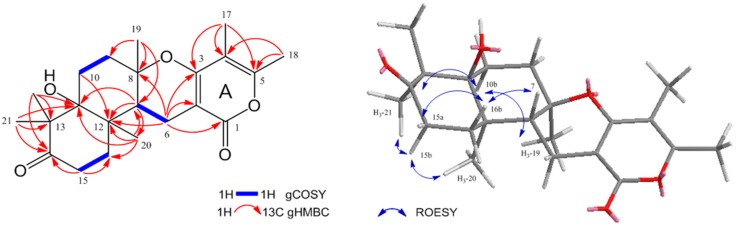
Key ^1^H-^1^H COSY, HMBC, and ROESY correlations of ochraceopone F (**1**).

Compound **1** was obtained as a dark brown oil and gave an [M + Na]^+^ ion at *m*/*z* 397.1987 (calcd. 397.1991) in the HR-ESI-MS (Figure S1), consistent with a molecular formula of C_22_H_30_O_5_Na. ^1^H (Figure S2) and ^13^C-NMR data (Table 1, Figure S3), and the COSY (Figure S4) and HSQC (Figure S5) spectra of **1** revealed the presence of one methine (*δ*_H_ 2.47), five methylenes (*δ*_H_ 1.68/2.01, 1.69/2.01, 1.91/2.18, 2.28, 2.47/2.64), six methyls (*δ*_H_ 1.12, 1.17, 1.21, 1.30, 1.88, 2.23), and ten quaternary carbons; one ketone carbonyl (*δ*_C_ 218.0) and one ester carbonyl (*δ*_C_ 165.7), two conjugated oxygenated enol carbons (*δ*_C_ 155.7, 163.9), two olefinic carbons (*δ*_C_ 97.6, 107.5), two oxygenated quaternary carbons (*δ*_C_ 80.4, 78.2), and two aliphatic quaternary carbons (*δ*_C_ 53.1, 40.4). Spin systems and their correlations were confirmed via COSY and HMBC correlations (Figure 2). The HMBC correlations (Figure S6) of two methyls (*δ*_H_ 1.88 and 2.23) and one methylene (*δ*_H_ 2.28) to the corresponding carbons, H_3_-17 (*δ*_H_ 1.88) to C-3 (*δ*_C_ 163.9), C-4 (*δ*_C_ 107.5), and C-5 (*δ*_C_ 155.7), H_3_-18 (*δ*_H_ 2.23) to C-4 and C-5, H-6 (*δ*_H_ 2.28) to C-1 (*δ*_C_ 165.7), C-2 (*δ*_C_ 97.6), and C-3, established the formation of an α-pyrone (A ring). The COSY correlations between H-6 and H-7 (*δ*_H_ 2.47), H-9 (*δ*_H_ 1.91, 2.18), and H-10 (*δ*_H_ 1.69, 2.01) and the HMBC correlations from H_3_-19 (*δ*_H_ 1.30) to C-7 (*δ*_C_ 42.9), C-8 (*δ*_C_ 80.4), and C-9 (*δ*_C_ 33.3) confirmed another ring formation (B ring) connected to the α-pyrone. The formation of 6-membered ring with the ketone (D ring) was also confirmed by the HMBC correlations from the protons of two germinal methyls H_3_-21 and 22 (*δ*_H_ 1.21 and 1.12, respectively) to C-11 (*δ*_C_ 78.2), C-13 (*δ*_C_ 53.1), and C-14 (*δ*_C_ 218.0) and from H-15 (*δ*_H_ 2.47, 2.64) to C-14 and C-16 (*δ*_C_ 32.4). The key HMBC correlation from H-20 (*δ*_H_ 1.17) to C-7, C-11, C-12, and C-16 established a sesquiterpene unit by connecting the AB ring system to the D ring. A database search of the tetracyclic ring of **1** identified a nearly similar natural product, ochraceopone E, an α-pyrone merosesquiterpenoid from the antarctic fungus *A. ochraceopetaliformis* [23]. The only difference between **1** and ochraceopone E is that **1** does not have a hydroxyl group at C-9. Thus, the structure of **1** was determined as 9-deoxy ochraceopone E and named ochraceopone F (**1**).

The relative configuration of **1** was established on the basis of ROESY spectrum (Figure 2 and Figure S7). The ROESY correlation from H_3_-19 (*δ*_H_ 1.30) to H_b_-10 (*δ*_H_ 2.01), H_b_-10 (*δ*_H_ 2.01) to H_3_-21 (*δ*_H_ 1.21), H_3_-21 (*δ*_H_ 1.21) to H_b_-15 (*δ*_H_ 2.64), and H_b_-15 (*δ*_H_ 2.64) to H_3_-20 (*δ*_H_ 1.17) placed these protons on the same face, while the absence of a ROESY correlation between two methyls (H_3_-19 and H_3_-20) and H-7 (*δ*_H_ 2.47) indicated that H-7, H_3_-19, and H_3_-20 were located on different faces at the ring junctions. Additionally, ROESY correlation from H-7 (*δ*_H_ 2.47) to H_b_-16 (*δ*_H_ 2.01) and H_b_-16 (*δ*_H_ 2.01) to H_a_-15 (*δ*_H_ 2.49) indicated that two methyls and the methine have different faces. The absolute configuration of ochraceopone E possessing an angular tetracyclic skeleton and having a very similar structure with **1** was determined by an X-ray diffraction study. Comparison of the optical rotation value, NMR chemical shifts, and ROESY correlations of **1** with those of ochraceopone E suggested that these compounds have the same absolute stereochemistry. This result was also supported by the fact that ochraceopones [19,23] share a biosynthetic pathway.

**Figure 3:**
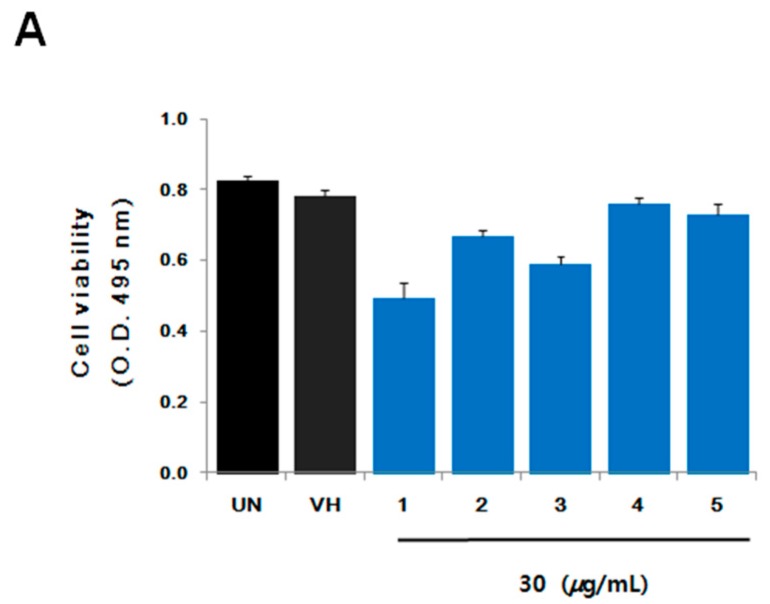

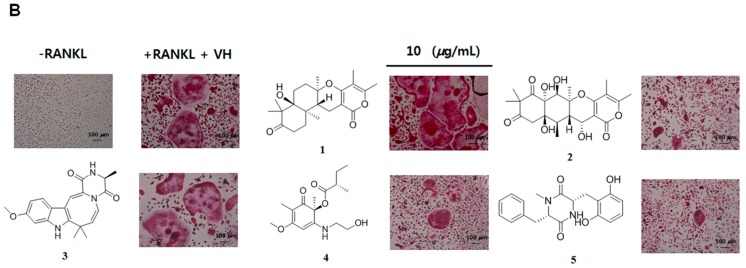
(**A**) Cell viability was measured by XTT assay. (**B**) Bone marrow macrophages (BMMs) were treated with vehicle or indicated concentration of Compounds **1**–**5** in the presence of RANKL and M-CSF. Cells were fixed with 10% formalin, stained by chromogenic substrate dissolved in tartate-containing buffer, and photographed under a light microscopy.

**Figure 4:**
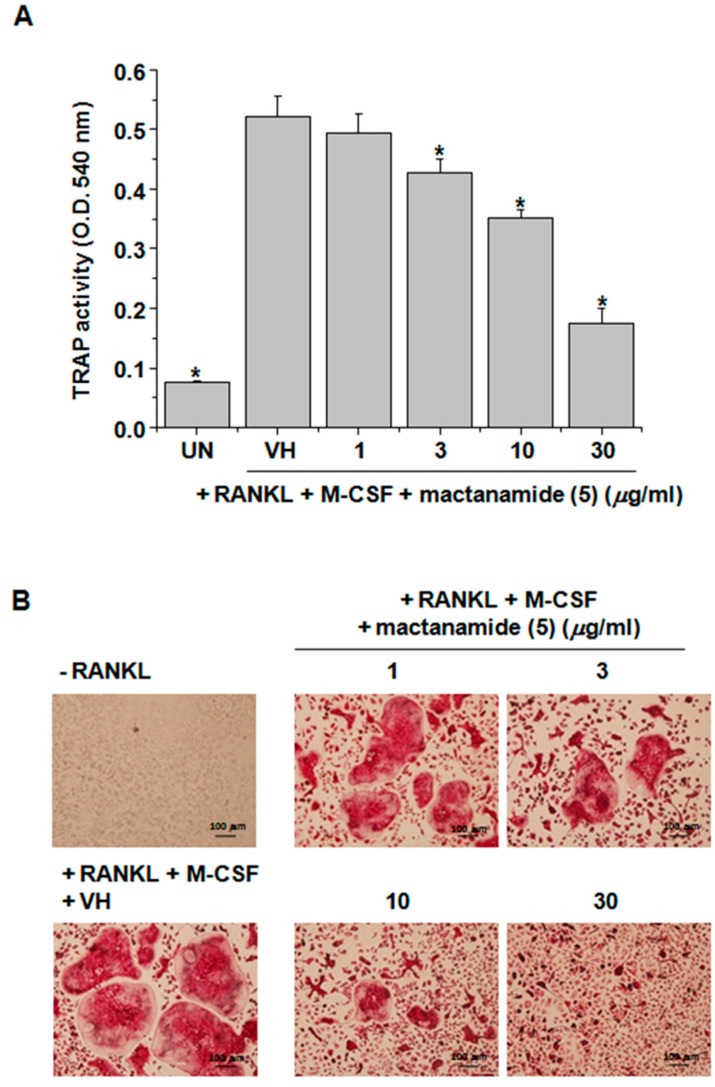
(**A**) Supernatants were mixed with chromogenic substrate dissolved in tartrate-containing buffer and TRAP activity was determined by measuring optical density at 540 nm. Each column shows the mean ± S.D. of quadruplicate determinations. Statistical significance was analyzed by one-way ANOVA and Dunnett’s *t*-test (* *p* < 0.05). (**B**) Cells were fixed with 10% formalin and stained by chromogenic substrate containing TRAP.

All the isolated compounds (**1**–**5**) were tested against human cancer cell lines using the sulforhodamine B (SRB) assay [24] and inhibitory effects on RANKL-induced osteoclast differentiation using tartate-resistant acid phosphatase (TRAP), which is highly expressed in osteoclasts, as a primary marker. These compounds did not show any cytotoxicity against five cancer cell lines (HCT 15, NUGC-3, NCI-H23, ACHN, PC-3, and MDA-MB-231) up to 30 μg/mL. However, aspertetranone D (**2**), wasabidienone E (**4**), and mactanamide (**5**) showed suppressive activities on RANKL-induced differentiation of BMMs into osteoclasts as evidenced by a TRAP assay (Figure 3B). To confirm cytotoxic effects on BMMs at their effective concentrations for the inhibition of differentiation of osteoclasts, the cell viability was measured by XTT assay (Figure 3A). Compounds **2**, **4**, and **5** had no significant cytotoxic effect on BMMs at concentrations used in this study. In particular, mactanamide (**5**) dose-dependently suppressed RANKL-induced differentiation of BMMs into osteoclasts (Figure 4A,B). These biological data indicate that mactanamide (**5**), displaying an inhibitory effect against osteoclastogenesis, might be a therapeutic drug lead for various skeletal diseases, and further research including mechanistic studies is needed.

## 3. Materials and Methods

### 3.1. General Experimental Procedures

1D (^1^H and ^13^C) and 2D (COSY, ROESY, HSQC, and HMBC) NMR spectra were acquired on a Varian Unity 500 MHz spectrometer (Varian Inc., Palo Alto, CA, USA). UV spectra were obtained on a Shimadzu UV-1650PC spectrophotometer (Shimadzu Corporation, Kyoto, Japan). IR spectra were recorded on a JASCO FT/IR-4100 spectrophotometer (JASCO Corporation, Tokyo, Japan). Optical rotations were measured on a Autopol III polarimeter (Rudolph Research Analytical, Hackettstown, NJ, USA). High-resolution ESIMS was recorded on a hybrid ion-trap time-of-flight mass spectrometer (SYNAPT G2, Wasters Corporation, Milford, CT, USA). HPLC was performed on a PrimeLine pump (Analytical Scientific Instrument, Inc., El Sobrante, CA, USA) with RI-101 (Shodex, Munich, Germany). Semi-preparative HPLC was performed using an ODS column (YMC-Pack-ODS-A, 250 × 10 mm i.d, 5 µm) (YMC Corporation, Kyoto, Japan). Analytical HPLC was conducted on an ODS column (YMC-Pack-ODS-A, 250 × 4.6 mm i.d, 5 µm) (YMC Corporation, Kyoto, Japan).

### 3.2. Fungal Material and Fermentation

The fungus *A. flocculosus* 01NT.1.1.5 was originally isolated from the sponge *Stylissa* sp. collected at Nha Trang Bay, Vietnam, in February 2016. The fungus was identified according to its gene sequences of 28S rRNA (GenBank accession number EU021616.1). A BLAST search result indicated that the sequence is similar 100% to the sequence of *A. flocculosus* (compared with NRRL 5224). In order to search for bioactive secondary metabolites of the strain, the fungus was grown stationary at 22 °C for 21 days in 100 Erlenmeyer flasks (500 mL), each containing 20 g of rice, 20 mg of yeast extract, 10 mg of KH_2_PO_4_, and 40 mL of natural sea water [25].

### 3.3. Isolation of Compounds

Strain 01NT-1.1.5 was grown stationary at 22 °C for 21 days in 100 Erlenmeyer flasks (500 mL), each containing 20 g of rice, 20 mg of yeast extract, 10 mg of KH_2_PO_4_, and 40 mL of natural sea water. The mycelia and medium were homogenized and extracted with EtOAc and then concentrated in vacuo to yield the crude extract (10 g). The crude extract was fractionated by flash column chromatography on ODS using a stepwise elution (each fraction 300 × 3) with combinations of MeOH/H_2_O (1:4, 2:3, 3:2, 4:1 and 100% MeOH). The second fraction eluted with MeOH/H_2_O (2:3) was purified by a semi-preparative reversed-phase HPLC (YMC-Pack-ODS-A, 250 × 10 mm i.d, 5 µm, flow rate 3.0 mL/min, RI detector) using isocratic elution with 22% ACN in H_2_O to yield Compound **4** (48.9 mg, t_R_ = 17 min). The third fraction eluted with MeOH/H_2_O (2:3) was subjected to a semi-preparative reversed-phase HPLC (YMC-Pack-ODS-A, 250 × 10 mm i.d, 5 µm, flow rate 3.0 mL/min, RI detector) using isocratic elution with 22% ACN in H_2_O to yield Compounds **2** (30.8 mg, t_R_ = 20 min) and **5** (4.9 mg, t_R_ = 44 min). The first fraction eluted with MeOH/H_2_O (3:2) was purified by a semi-preparative reversed-phase HPLC (YMC-Pack-ODS-A, 250 × 10 mm i.d, 5 µm, flow rate 4.0 mL/min, RI detector) using isocratic elution with 50% MeOH in H_2_O to yield Compound **3** (6.7 mg, t_R_ = 20 min). The third fraction eluted with MeOH/H_2_O (3:2) was subjected to an analytical reversed-phase HPLC (YMC-Pack-ODS-A, 250 × 4.6 mm i.d, 5 µm, flow rate 2.0 mL/min, RI detector) using isocratic elution with 50% ACN in H_2_O to obtain seven compounds. Among the compounds, the first compound was purified by a subsequent analytical reversed-phase HPLC (YMC-Pack-ODS-A, 250 × 4.6 mm i.d, 5 µm, flow rate 2.0 mL/min, RI detector) using isocratic elution with 45% ACN in H_2_O to yield pure Compound **1** (5.2 mg, t_R_ = 7 min).

Ochraceopone F (**1**): dark brown oil; ${\left[\alpha \right]}_{D}^{25}$ −10.0(c 1.0, MeOH); IR ν_max_ 3303, 2360, 2332, 1706, 1646, 1282, 1186 cm^−1^; UV(MeOH) λ_max_ (log ε) 348 (3.93), 264 (4.18), 224 (4.57) nm; HR-ESI-MS *m*/*z* 397.1987 [M + Na]^+^ (calcd. for 397.1991, C_22_H_30_O_5_Na); ^1^H NMR (CD_3_OD, 500 MHz) and ^13^C-NMR (CD_3_OD, 125 MHz) see Table 1.

### 3.4. Cell Viability Assay

For cell viability assay, BMMs (4 × 10^4^ cells/well) were cultured in the presence of M-CSF (50 ng/mL) in 96-well plates with or without compounds (**1**–**5**). After 4 days, the XTT assay was measured using Cell Proliferation Assay Kit II (Roche Applied Science, Penzberg, Germany). In brief, the XTT labeling mixture was prepared by mixing 50 volumes of 1 mg/mL sodium 3′-[1-(phenylaminocrbony)-3,4-tetrazolium]-bis(4-methoxy-6-nitro) benzene sulfonic acid hydrate (in RPMI 1640) with 1 volume of 0.383 mg/mL of *N*-methyldibenzopyrazine methyl sulfate (in PBS). This XTT labeling mixture was added to the cultures and incubated for 2 h at 37 °C. Absorbance was measured at 495 nm with a reference wavelength at 650 nm.

### 3.5. Osteoclastogenesis Assay

Mouse bone marrow cells were isolated from femurs and tibiae of 6~8 weeks old female C57BL/6 mice (Koatech, Pyungtaek, Gyeonggi, Korea). After lysing red blood cells, cells were incubated in minimal essential medium (Gibco BRL, Gaithersburg, MD, USA) supplemented with 10% fetal bovine serum, 100 U/mL penicillin, and 100 g/mL streptomycin in the presence of M-CSF (50 ng/mL) for 3 days. BMMs were obtained by removing floating cells. For osteoclast differentiation, BMMs (4 × 10^4^ cells/well) were cultured in the presence of M-CSF (50 ng/mL) and RANKL (100 ng/mL) in 96-well plates with or without compounds (**1**–**5**). After 4 days, cells were fixed with 10% formalin for 5 min, stained for TRAP-positive cells, and photographed under a light microscopy. Quantitation of TRAP activity in culture supernatants was performed using TRAP staining kit (Kamiya Biomedical Company, Tukwila, WA, USA) according to the manufacturer’s instructions.

## 4. Conclusions

We have isolated a new α-pyrone merosesquiterpenoid containing an angular tetracyclic carbon skeleton, ochraceopone F (**1**), and four known compounds, aspertetranone D (**2**), cycloechinulin (**3**), wasabidienone E (**4**), and mactanamide (**5**). All compounds had no cytotoxic effect on human cancer cell lines (HCT 15, NUGC-3, NCI-H23, ACHN, PC-3, and MDA-MB-231) up to 30 μg/mL. Aspertetranone D (**2**) and wasabidienone E (**4**) had a weak osteoclast differentiation inhibitory activity. Interestingly, mactanamide (**5**) displayed a potent suppression effect of osteoclast differentiation without any evidence of cytotoxicity at the effective concentrations. Mactanamide (**5**), a new fungistatic diketopiperazine containing the uncommon amino acid d-2,6-dihydroxyphenylalanine, has been first isolated from the mycelium of an undescribed marine fungus of the genus *Aspergillus* sp. from the surface of the brown marine alga *Sargassum* sp. [22]. However, to the best of our knowledge, this is the first report to describe the suppressive effect of mactanamide on osteoclast differentiation. This study revealed that mactanamide may have a significant role in the inhibition of osteoclast differentiation. However, to address the potential therapeutic efficacy, further research including mechanism of action study is needed.

## Figures and Tables

**Table 1:** ^1^H (500 MHz) and ^13^C-NMR (125 MHz) data for **1** in CD_3_OD.

Position	1			
	*δ*H (*J*, Hz)	*δ*_C_	HMBC	COSY
1	-	165.7		
2	-	97.6		
3	-	163.9		
4	-	107.5		
5	-	155.7		
6	2.28, dd (16.5, 5.0)	16.5	1, 2, 3, 8, 12	7
7	2.47, dd (13.0, 5.0)	42.9	11, 12, 20	6
8		80.4		
9a	1.91, brt	33.3		10
9b	2.18, overlap			10
10a	1.69, overlap	24.5	12	9
10b	2.01, overlap		12	9
11	-	78.2		
12	-	40.4		
13	-	53.1		
14	-	218.0		
15a	2.49, m	33.3	14, 16	16
15b	2.64, m		14, 16	16
16a	1.68, overlap	32.4		15
16b	2.01, overlap			15
17	1.88, s	8.1	3, 4, 5	
18	2.23, s	15.7	4, 5	
19	1.30, s	19.3	7, 8, 9	
20	1.17, s	17.3	7, 11, 12, 16	
21	1.21, s	22.5	11, 13, 14, 22	
22	1.12, s	20.9	11, 13, 14, 21	

## Supplementary Materials

The following are available online at www.mdpi.com/1660-3397/16/1/14/s1. Figures S1–S7: HR-ESI-MS data and ^1^H NMR, ^13^C-NMR, ^1^H-^1^H COSY, HSQC, HMBC, and ROESY spectra of **1**; Figures S8–S19: LRMS data and ^1^H NMR and ^13^C-NMR spectra of **2**–**5**.
