# Supplementary material for: Suppression of RANKL-Induced Osteoclastogenesis by the Metabolites from the Marine Fungus Aspergillus flocculosus Isolated from a Sponge Stylissa sp
Source: Mar Drugs. 2018 Jan 5;16(1):14. doi: 10.3390/md16010014 (PMC5793062; doi:10.3390/md16010014)
Supplement: Supplementary file 1 [file marinedrugs-16-00014-s001.pdf]

Supplementary data

Suppression of RANKL-Induced Osteoclastogenesis by the Metabolites from the Marine Fungus *Aspergillus flocculosus* Isolated from a Sponge *Stylissa* sp.

Hee Jae Shin[1,2,*], Byeoung-Kyu Choi[1,2], Phan Thi Hoai Trinh[4,5], Hwa-Sun Lee[1], Jong Soon Kang[3], Tran Thi Thanh Van[4,5], Hyi-Seung Lee[1], Jong Seok Lee[1], Yeon-Ju Lee[1], and Jihoon Lee[1]

[1]*Marine Natural Products Laboratory, Korea Institute of Ocean Science & Technology, 385, Haeyang-ro, Yeongdo-gu, Busan Metropolitan City 49111, Republic of Korea*

[2]*Department of Marine Biotechnology, University of Science and Technology, 217, Gajeong-ro, Yuseong-gu, Daejeon 34113, Korea*

[3]*Bio-Evaluation Center, Korea Research Institute of Bioscience and Biotechnology, 30 Yeongudanjiro, Cheongju 28116, Republic of Korea*

[4]*Nhatrang Institute of Technology Research and Application, Vietnam Academy of Science and Technology, 02 Hung Vuong, Nha Trang 650000, Vietnam*

[5]*Graduate University of Science and Technology, Vietnam Academy of Science and Technology, 18 Hoang Quoc Viet, Cau Giay, Ha Noi 100000, Vietnam*

Contents

1. Experimental section. --1-2

2. Spectral information of compounds (**1**-**5**).
Figure S1. HRESI-MS data of ochraceopone F (**1**). ---3
Figure S2. ^{1}H NMR spectrum of ochraceopone F (**1**). --4
Figure S3. ^{13}C NMR spectrum of ochraceopone F (**1**). ---5
Figure S4. ^{1}H-^{1}H COSY spectrum of ochraceopone F (**1**). --6
Figure S5. HSQC spectrum of ochraceopone F (**1**). --7
Figure S6. HMBC spectrum of ochraceopone F (**1**).--8
Figure S7. ROESY spectrum ochraceopone F (**1**). ---9
Figure S8. LRMS data of aspertetranone D (**2**). -- 10
Figure S9. ^{1}H NMR spectrum of aspertetranone D (**2**). -- 11
Figure S10. ^{13}C NMR spectrum of aspertetranone D (**2**). -- 12
Figure S11. LRMS data of cycloechinulin (**3**). --- 13
Figure S12. ^{1}H NMR spectrum of cycloechinulin (**3**).--- 14
Figure S13. ^{13}C NMR spectrum of cycloechinulin (**3**). --- 15
Figure S14. LRMS data of wasabidienone E (**4**).--- 16
Figure S15. ^{1}H NMR spectrum of wasabidienone E (**4**).-- 17
Figure S16. ^{13}C NMR spectrum of wasabidienone E (**4**). -- 18
Figure S17. LRMS data of mactanamide (**5**). -- 19
Figure S18. ^{1}H NMR spectrum of mactanamide (**5**). --- 20
Figure S19. ^{13}C NMR spectrum of mactanamide (**5**). -- 21

General Experimental Procedures. 1D (^{1}H and ^{13}C) and 2D (COSY, ROESY, HSQC, and HMBC) NMR spectra were acquired on a Varian Unity 500 MHz spectrometer. UV spectra were obtained on a Shimadzu UV-1650PC spectrophotometer. IR spectra were recorded on a JASCO FT/IR-4100 spectrophotometer. Optical rotations were measured on a JASCO (DIP-1000) digital polarimeter. High-resolution ESIMS was recorded on a hybrid ion-trap time-of-flight mass spectrometer (Shimadzu LC/MS-IT-TOF). HPLC was performed on a PrimeLine Binary pump with RI-101(Shodex). Semi-preparative HPLC was performed using an ODS column (YMC-Pack-ODS-A, 250 × 10 mm i.d, 5 μm). Analytical HPLC was conducted on an ODS column (YMC-Pack-ODS-A, 250 × 4.6 mm i.d, 5 μm).

Isolation of compounds 1-5. Strain 01NT-1.1.5 was grown stationary at 22℃ for 21 days in 100 Erlenmeyer flasks (500mL), each containing 20g of rice, 20 mg of yeast extract, 10 mg of KH_2PO_4, and 40 mL of natural sea water. The mycelia and medium were homogenized and extracted with EtOAc and then concentrated in vacuo to yield the crude extract (10 g). The crude extract was fractionated by flash column chromatography on ODS using a stepwise elution (each fraction 300× 3) with combinations of MeOH/H_2O (1:4, 2:3, 3:2, 4:1 and 100% MeOH). The second fraction eluted with MeOH/H_2O (2:3) was purified by a semi-preparative reversed-phase HPLC (YMC-Pack-ODS-A, 250 × 10 mm i.d, 5 μm, flow rate 3.0 mL/min, RI detector) using isocratic elution with 22% ACN in H_2O to yield compound **4** (48.9mg, t_R = 17min) . The third fraction eluted with MeOH/H_2O (2:3) was subjected to a semi-preparative reversed-phase HPLC (YMC-Pack-ODS-A, 250 × 10 mm i.d, 5 μm, flow rate 3.0 mL/min, RI detector) using isocratic elution with 22% ACN in H_2O to yield compounds **2** (30.8mg, t_R = 20min) and **5** (4.9mg, t_R = 44min). The first fraction eluted with MeOH/H_2O (3:2) was purified by a semi-preparative reversed-phase HPLC (YMC-Pack-ODS-A, 250 × 10 mm i.d, 5 μm, flow rate 4.0 mL/min, RI detector) using isocratic elution with 50% MeOH in H_2O to yield compound **3** (6.7mg, t_R = 20min). The third fraction eluted with MeOH/H_2O (3:2) was subjected to an analytical reversed-phase HPLC (YMC-Pack-ODS-A, 250 × 4.6 mm i.d, 5 μm, flow rate 2.0 mL/min, RI detector) using isocratic elution with 50% ACN in H_2O to obtain seven compounds. Among the compounds, the first compound was purified by a subsequent analytical reversed-phase HPLC(YMC-Pack-ODS-A, 250 × 4.6 mm i.d, 5 μm, flow rate 2.0 mL/min, RI detector) using isocratic elution with 45% ACN in H_2O to yield pure compound **1** (5.2mg, t_R = 7min).

Ochraceopone F (**1**): colorless oil; $[\alpha]_D^{25}$ -10.0(c 1.0, MeOH); IR ν_{max} 3303, 2360, 2332, 1706, 1646, 1282, 1186 cm^{-1}; UV(MeOH) λ_{max} 348, 264, 224nm; HRESIMS m/z 397.1987 $[M + Na]^+$ (calcd for 397.1991, $C_{22}H_{30}O_5Na$); ^{1}H NMR (CD_3OD, 500MHz) and ^{13}C NMR (CD_3OD, 125 MHz) see **Table 1**.

Osteoclastogenesis assay. Mouse bone marrow cells were isolated from femurs and tibiae of 6~8

weeks old female C57BL/6 mice (Koatech, Pyungtaek, Gyeonggi, Korea). After lysing red blood cells, cells were incubated in minimal essential medium (Gibco BRL, MD, USA) supplemented with 10% fetal bovine serum, 100 U/mL penicillin and 100 g/mL streptomycin in the presence of M-CSF (50 ng/mL) for 3 days. BMMs were obtained by removing floating cells. For osteoclast differentiation, BMMs (4 x 10^4 cells/well) were cultured in the presence of M-CSF (50 ng/mL) and RANKL (100 ng/mL) in 96-well plates with or without compounds (**1-5**). After 4 days, cells were fixed with 10% formalin for 5 min, stained for TRAP-positive cells and photographed under a light microscopy. Quantitation of TRAP activity in culture supernatants was performed using TRAP staining kit (Kamiya Biomedical Company, WA, USA) according to manufacturer's instructions.

Elemental Composition Report

Single Mass Analysis
Tolerance = 5.0 PPM / DBE: min = -1.5, max = 50.0
Element prediction: Off
Number of isotope peaks used for i-FIT = 3

Monoisotopic Mass, Even Electron Ions
33 formula(e) evaluated with 1 results within limits (all results (up to 1000) for each mass)
Elements Used:
C: 1-30 H: 1-40 O: 1-10 Na: 1-1

Minimum:				-1.5				
Maximum:		100.0	5.0	50.0				
Mass	Calc. Mass	mDa	PPM	DBE	i-FIT	Norm	Conf(%)	Formula
397.1987	397.1991	-0.4	-1.0	7.5	719.9	n/a	n/a	C22 H30 O5 Na

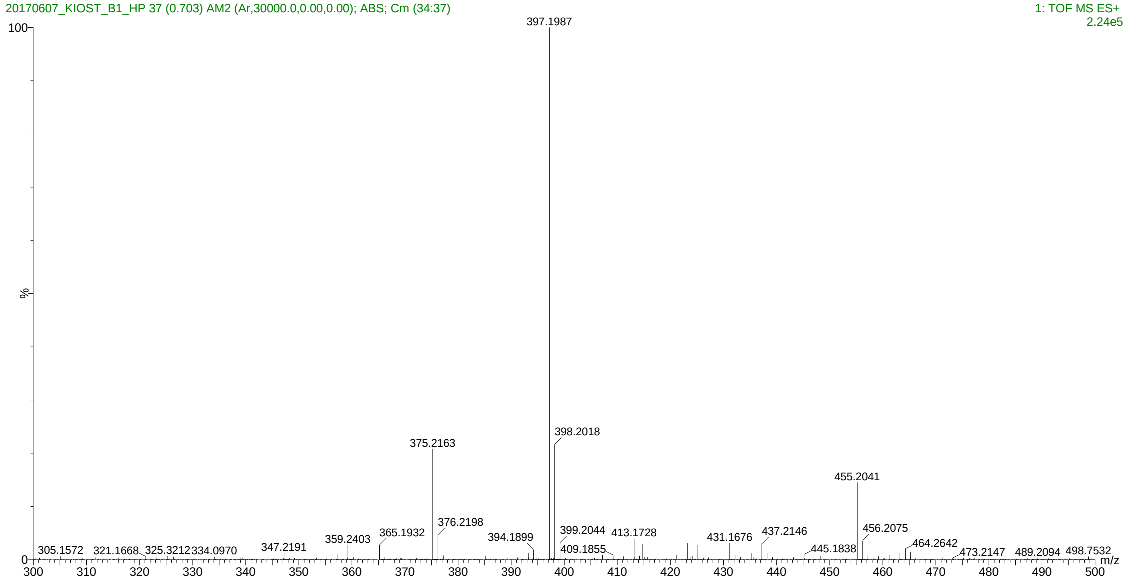

Figure S1. HRESIMS data of ochraceopone F (**1**).

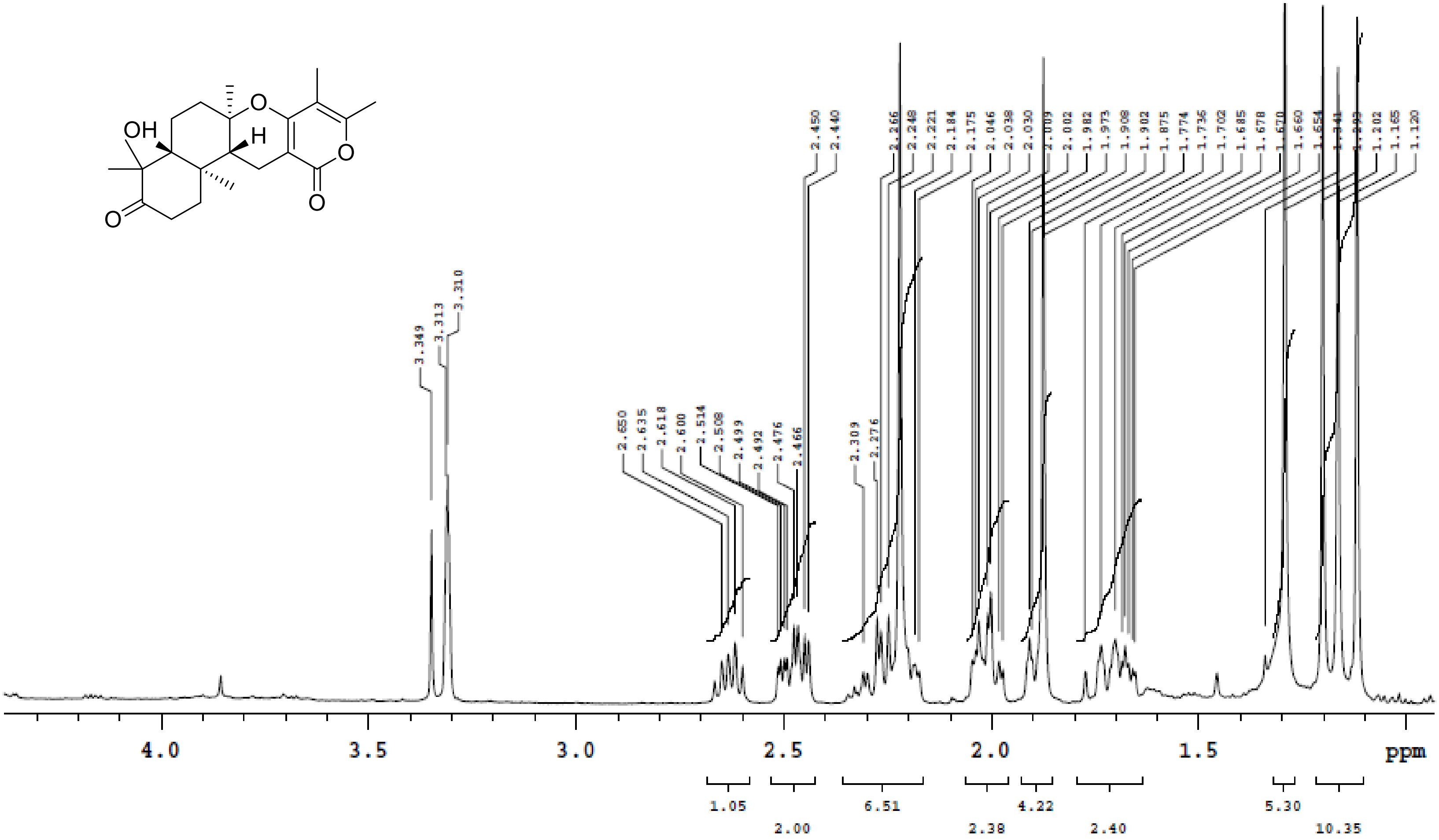

Figure S2. ^{1}H NMR spectrum of ochraceopone F (**1**).

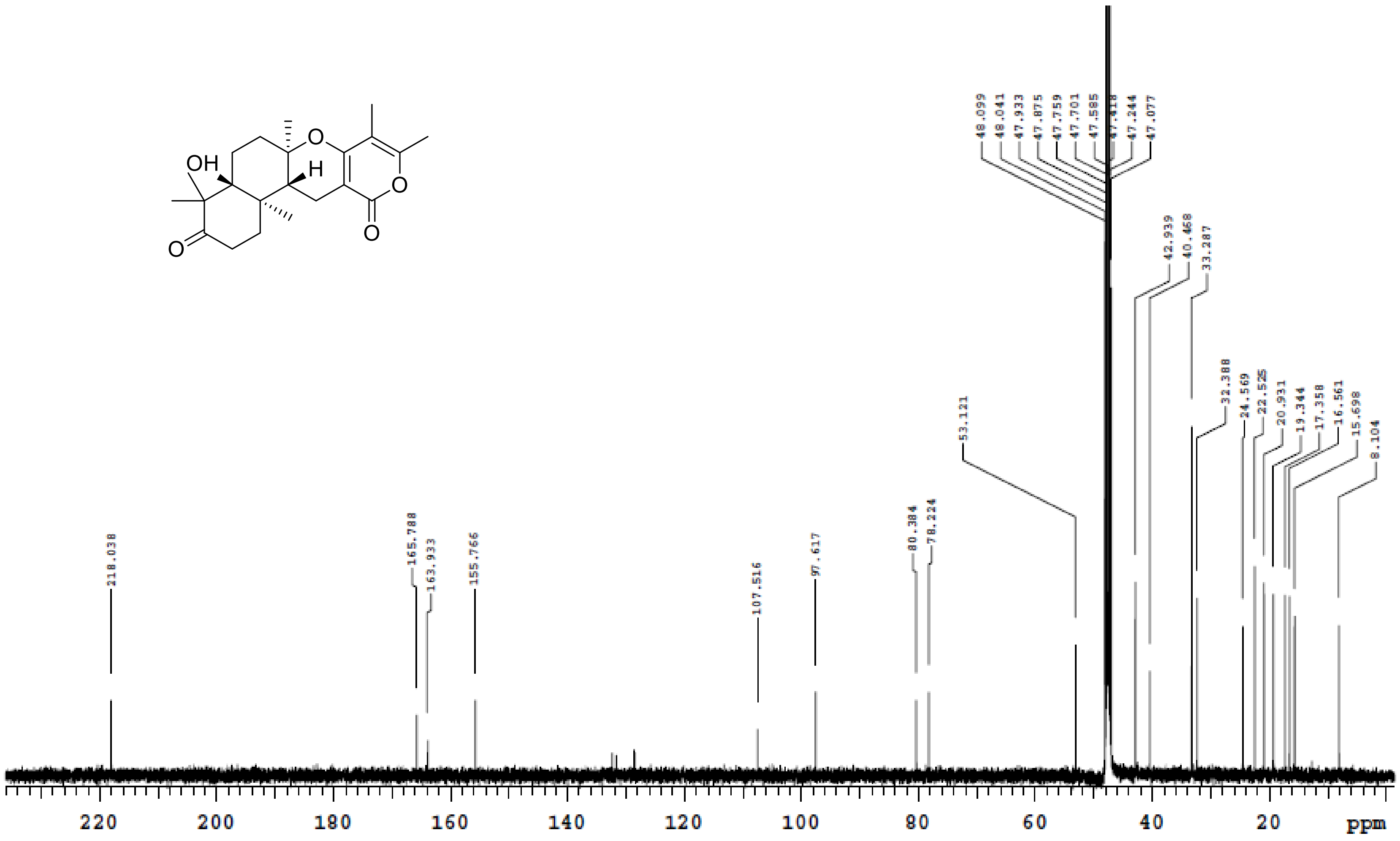

Figure S3. ^{13}C NMR spectrum of ochraceopone F (**1**).

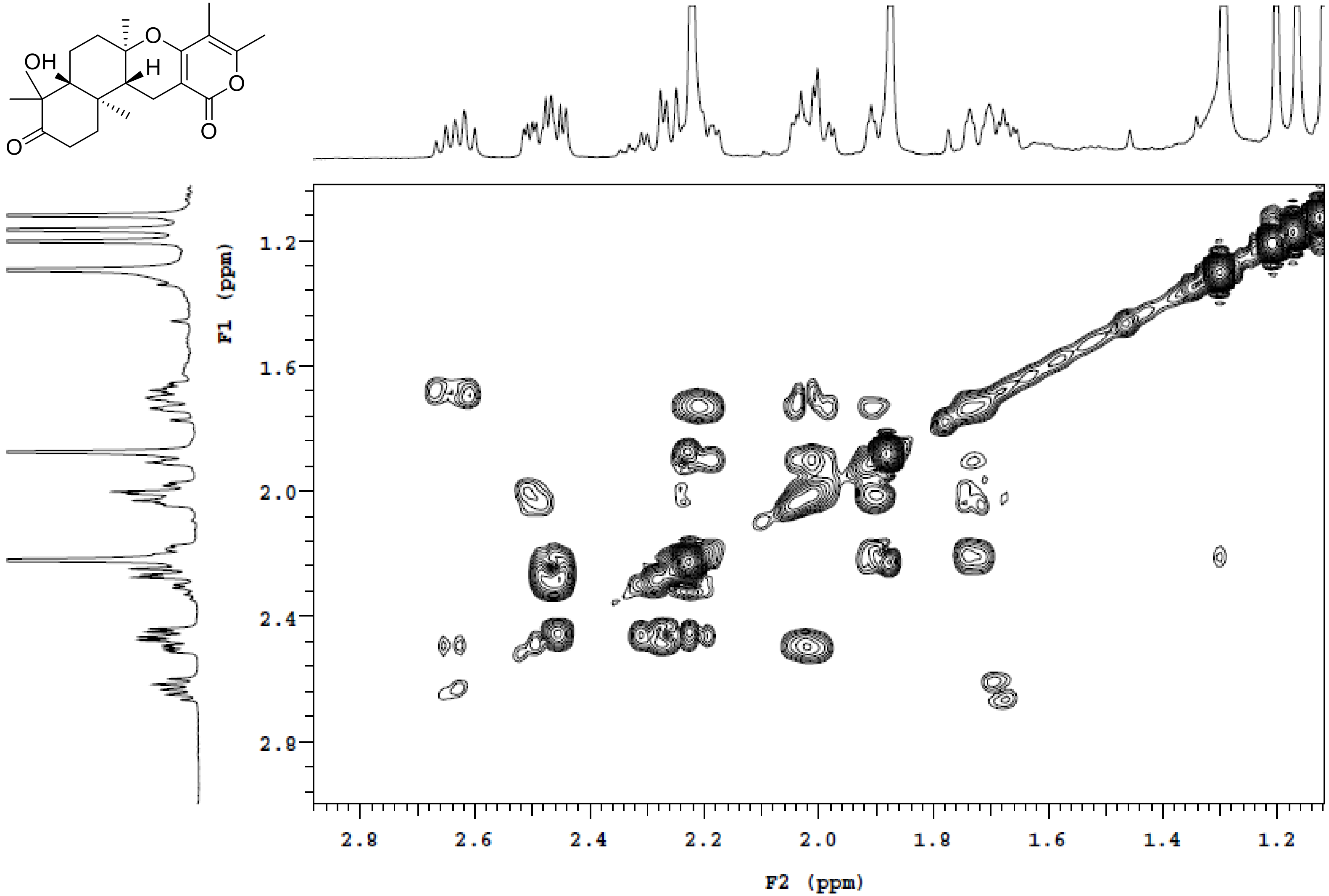

Figure S4. ^{1}H-^{1}H COSY spectrum of ochraceopone F (**1**).

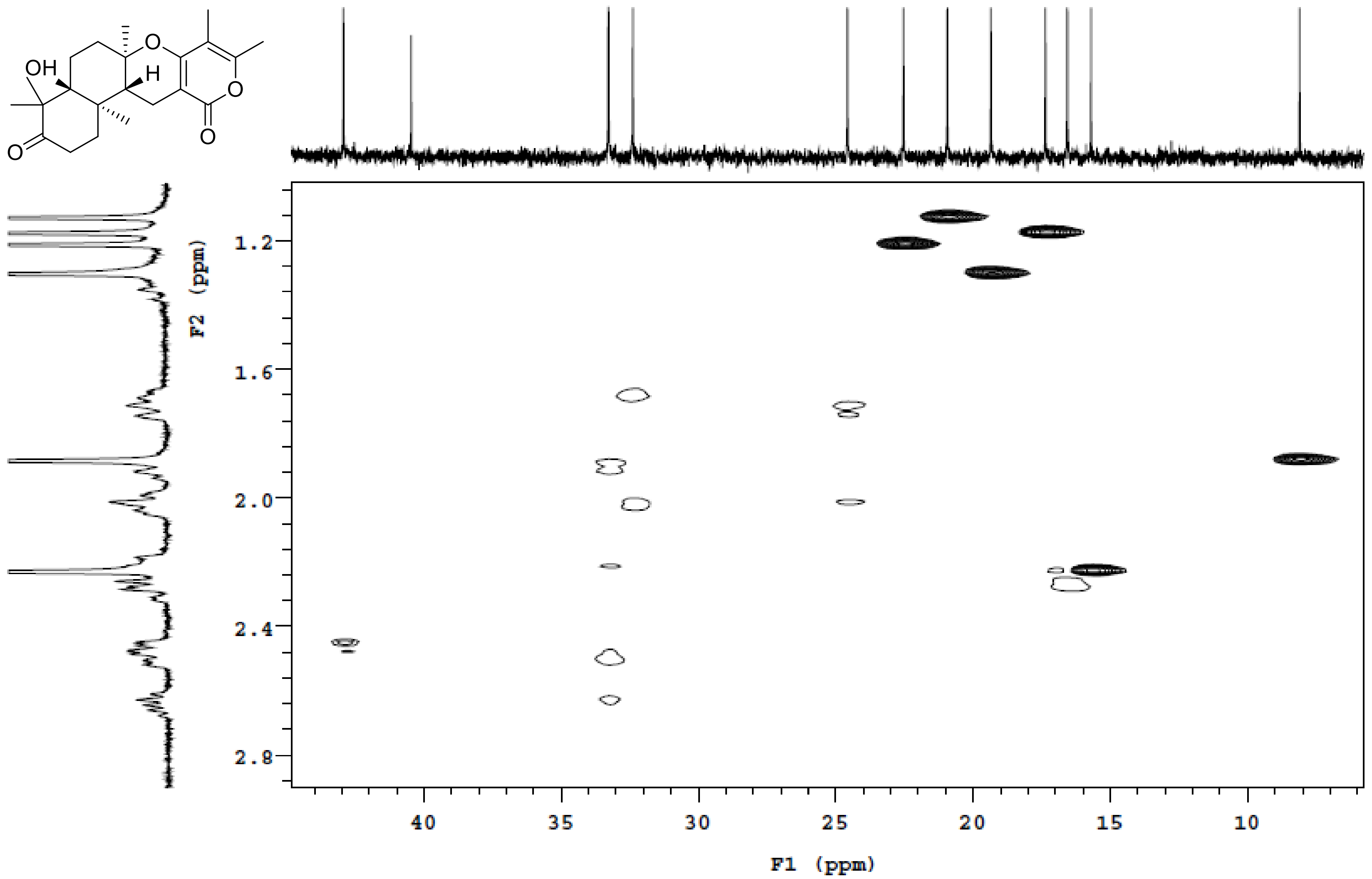

Figure S5. HSQC spectrum of ochraceopone F (**1**).

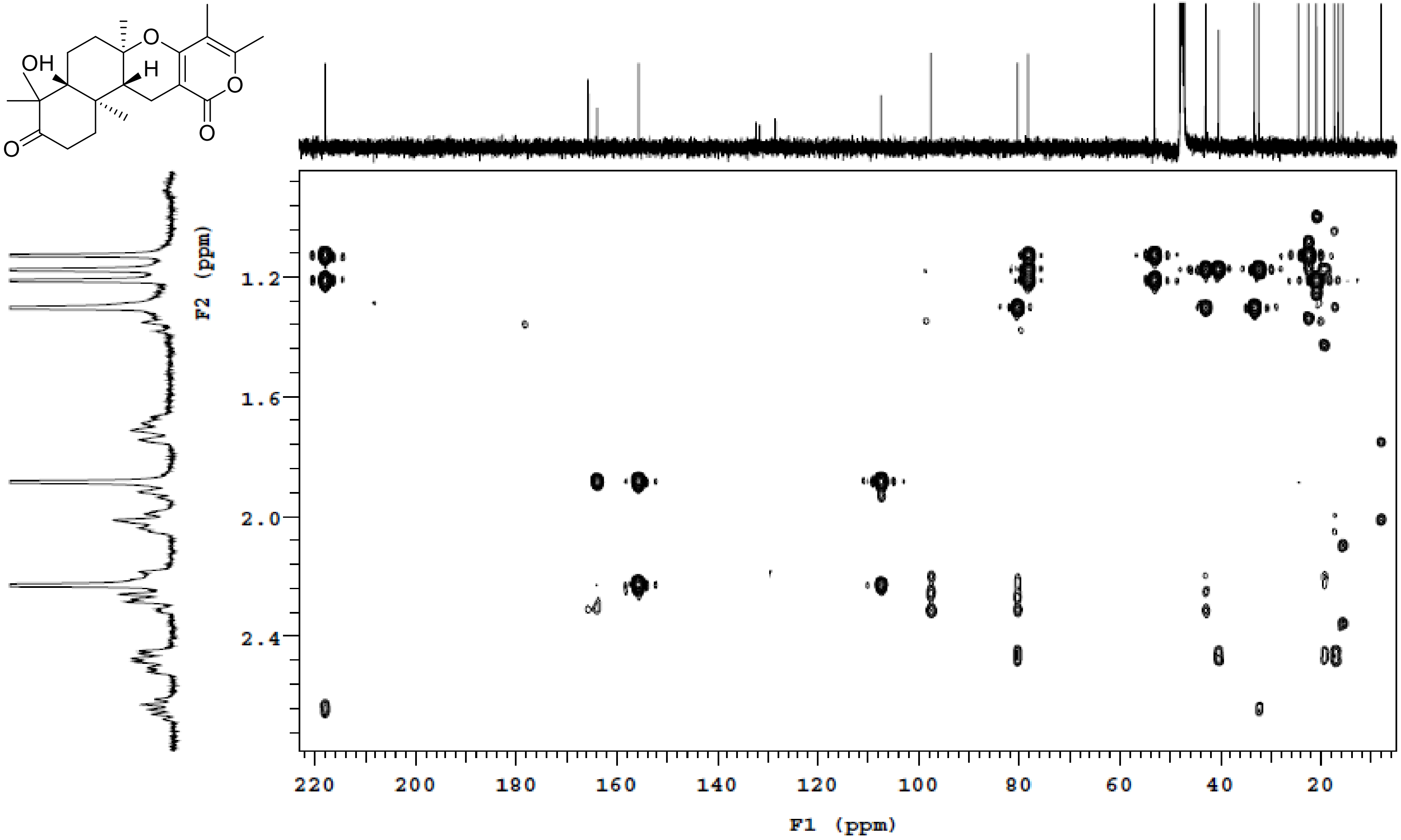

Figure S6. HMBC spectrum of ochraceopone F (**1**).

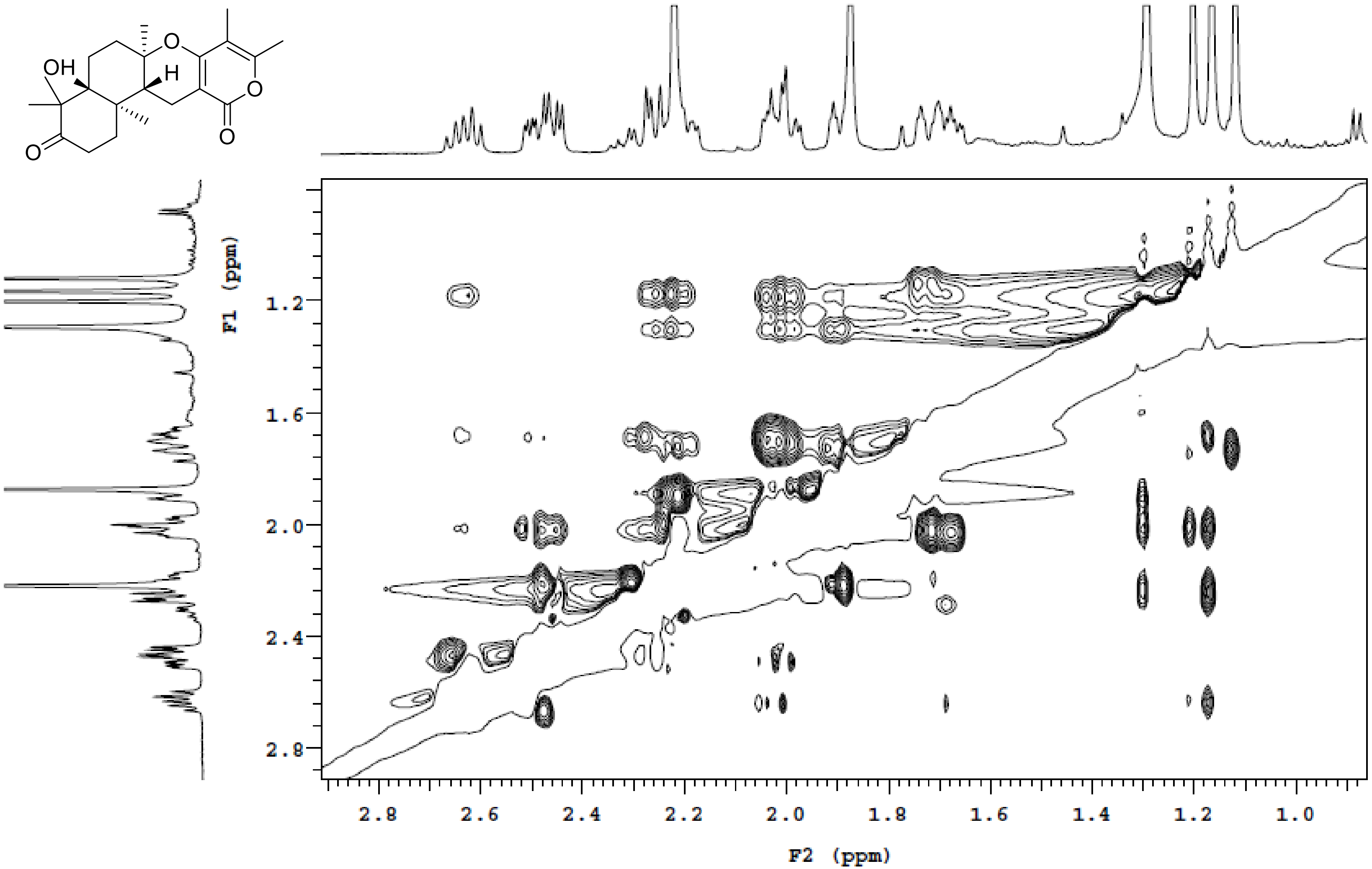

Figure S7. ROESY spectrum ochraceopone F (**1**).

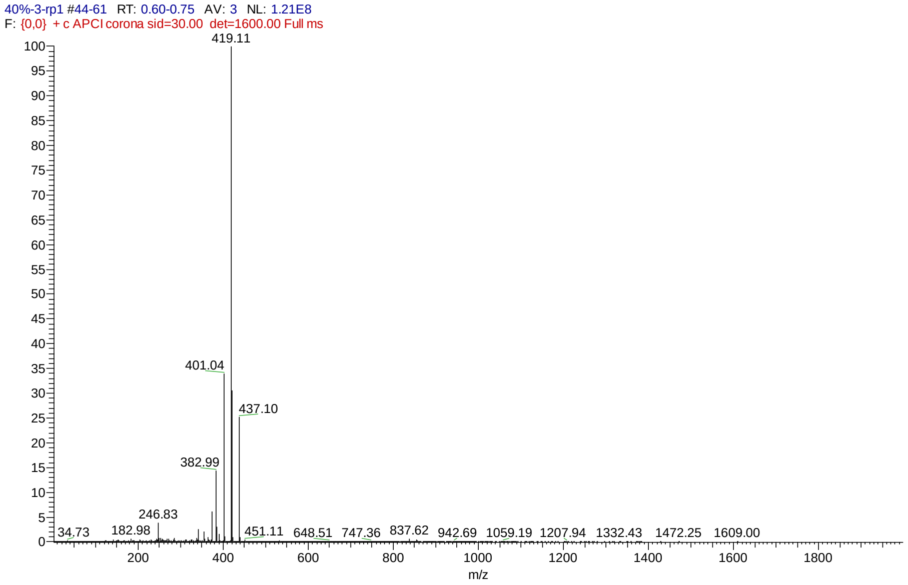

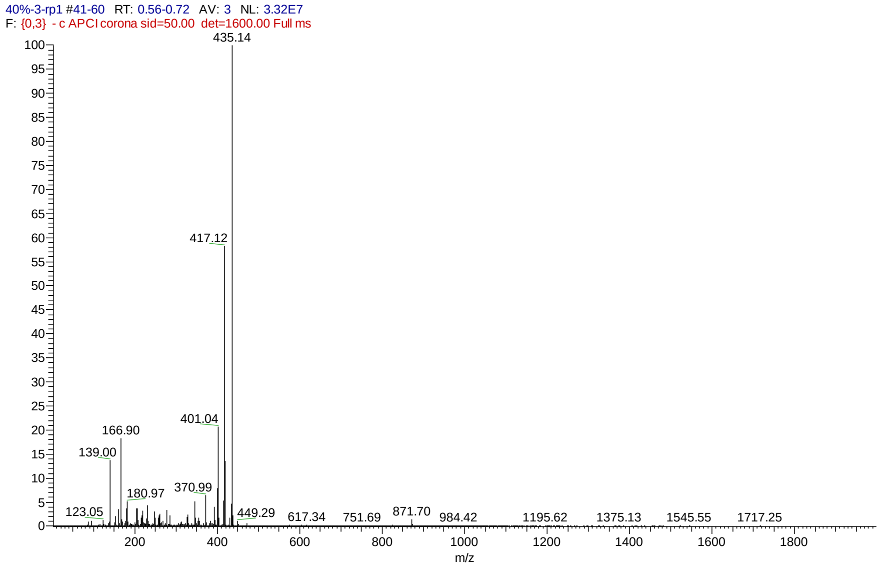

Figure S8. LRMS data of aspertetranone D (**2**).

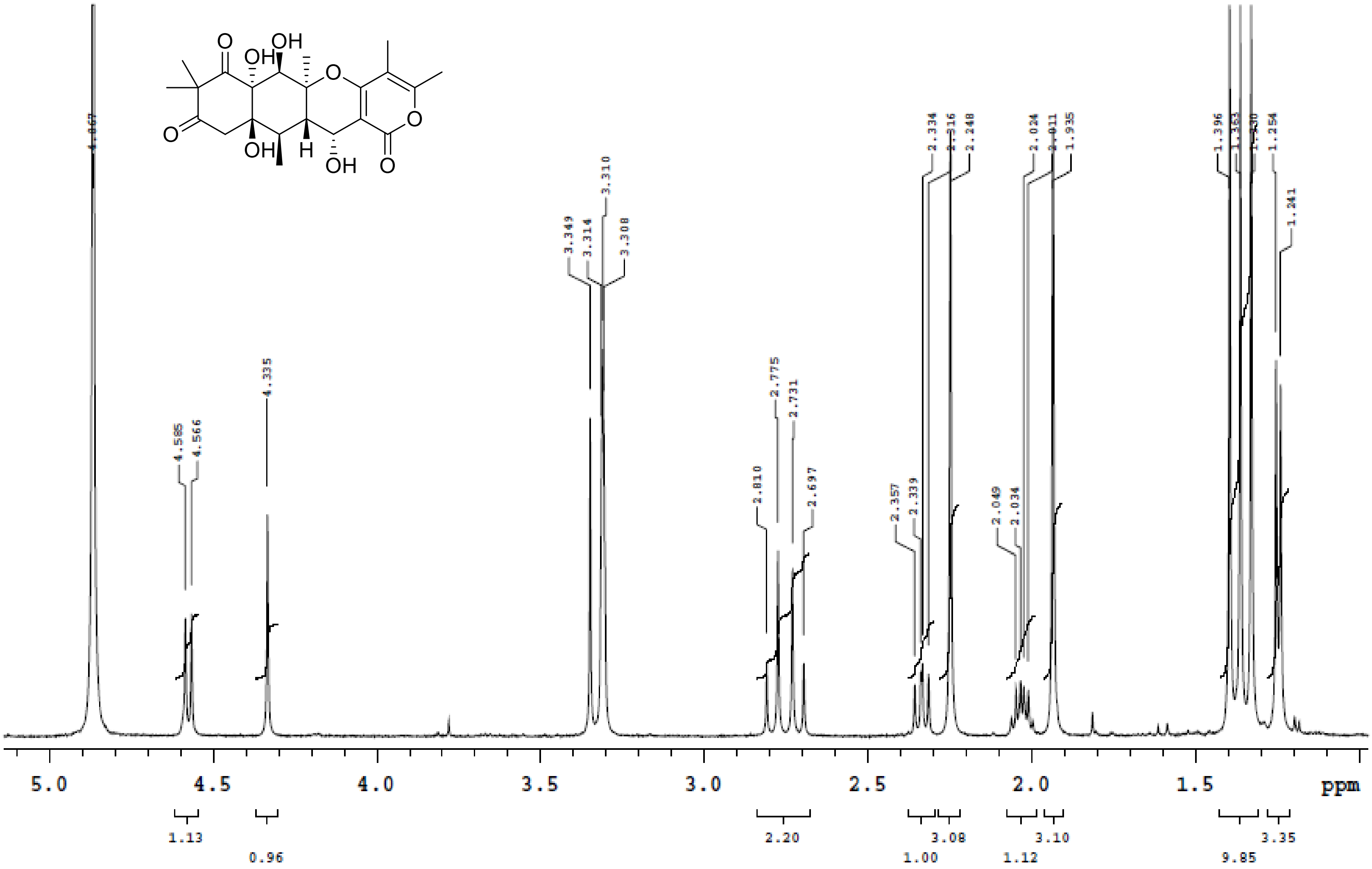

Figure S9. ^{1}H NMR spectrum of aspertetranone D (**2**).

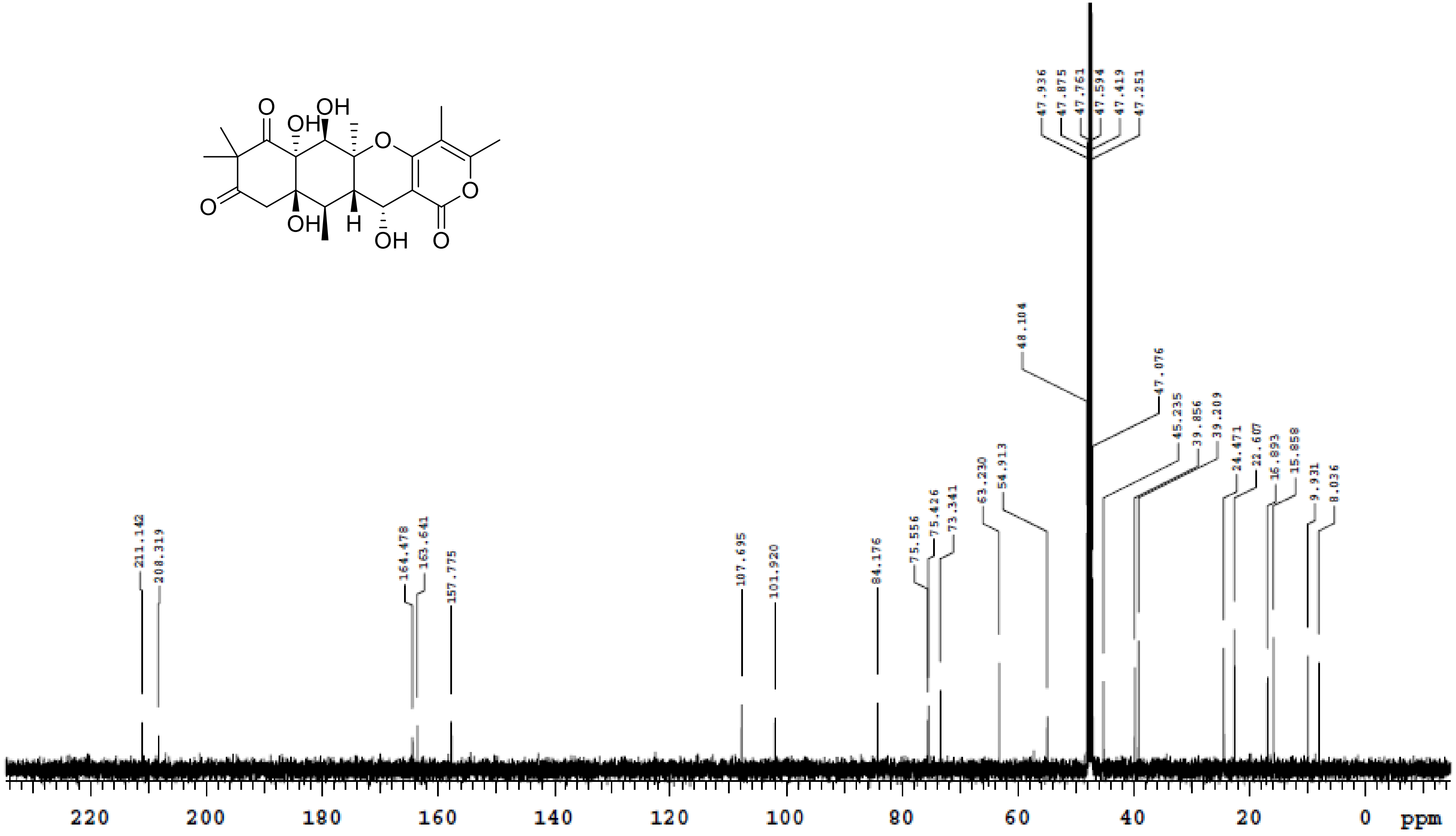

Figure S10. ^{13}C NMR spectrum of aspertetranone D (**2**).

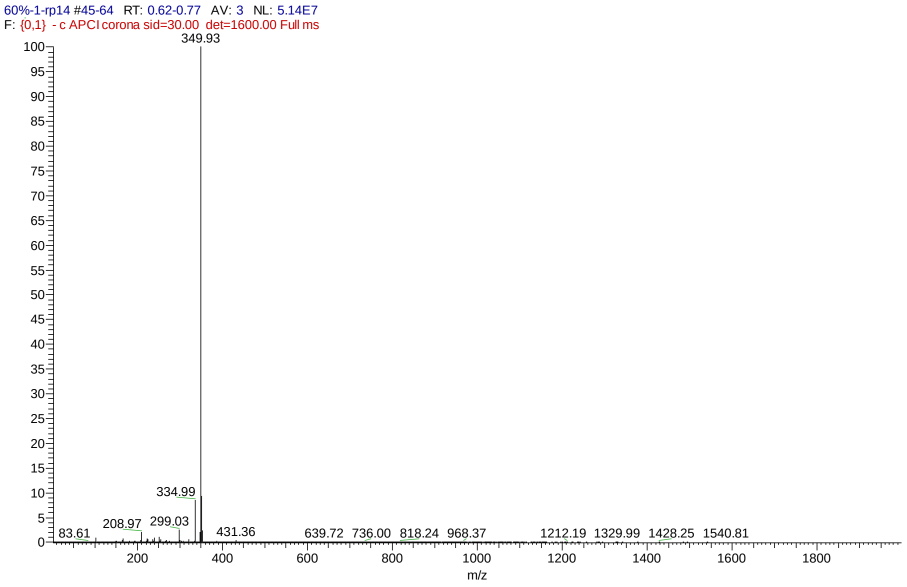

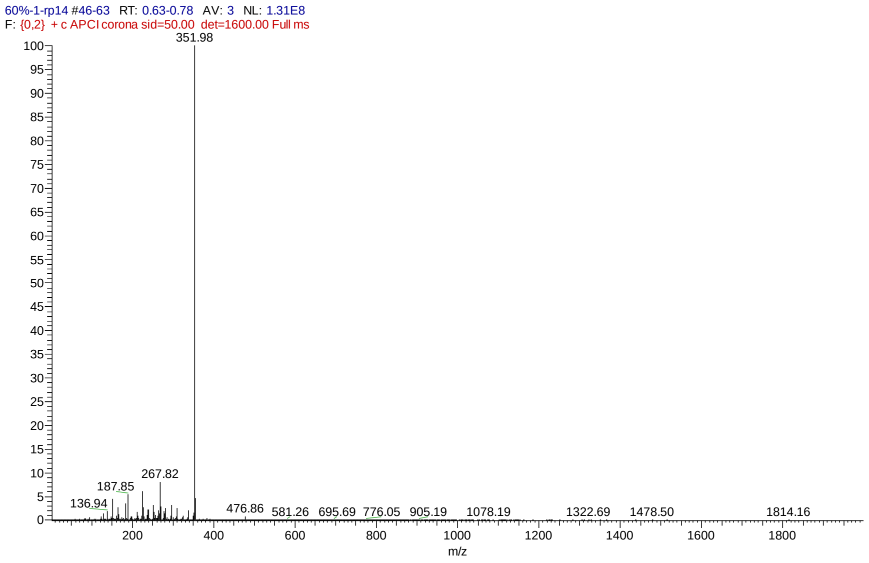

Figure S11. LRMS data of cycloechinulin (**3**).

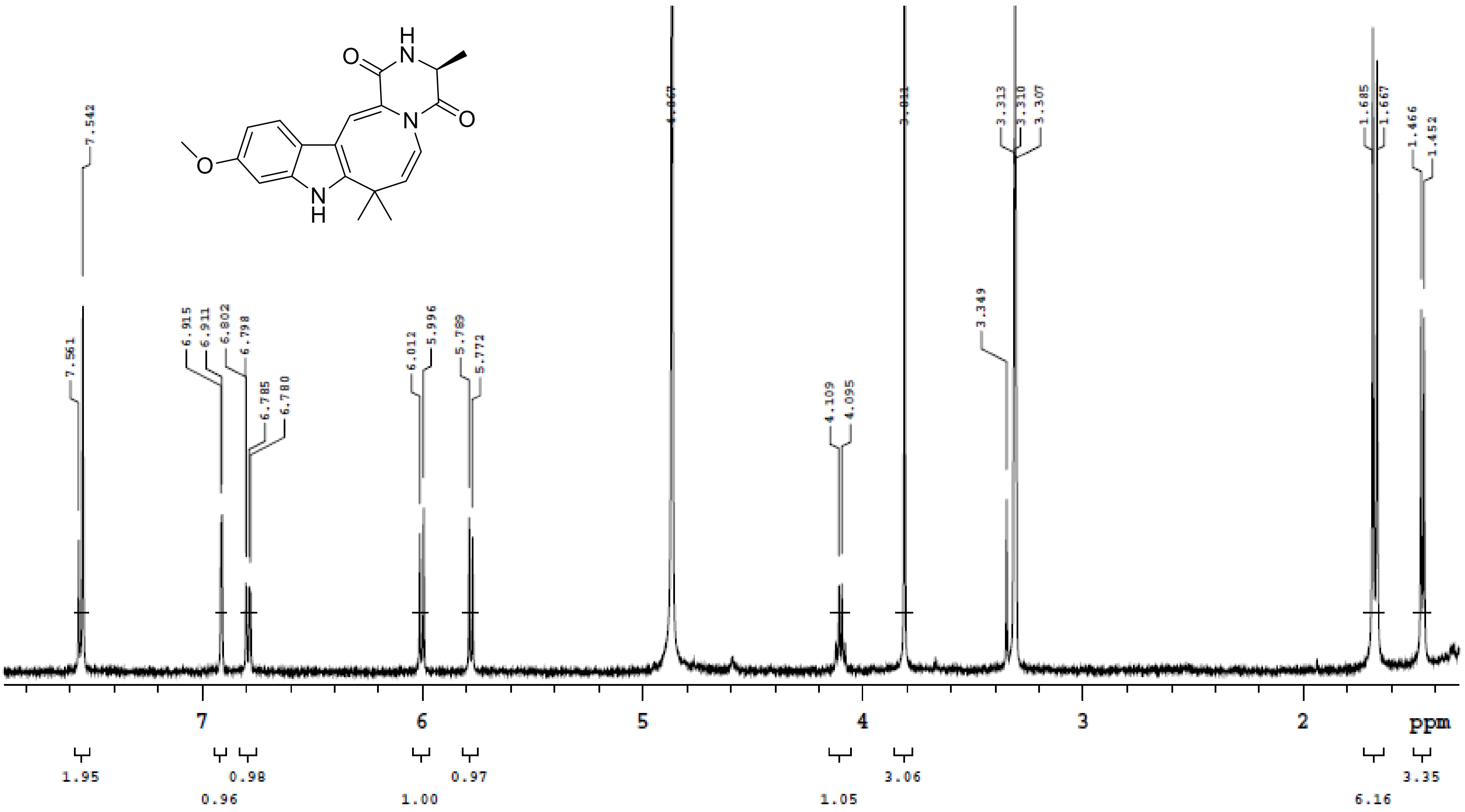

Figure S12. ^{1}H NMR spectrum of cycloechinulin (**3**).

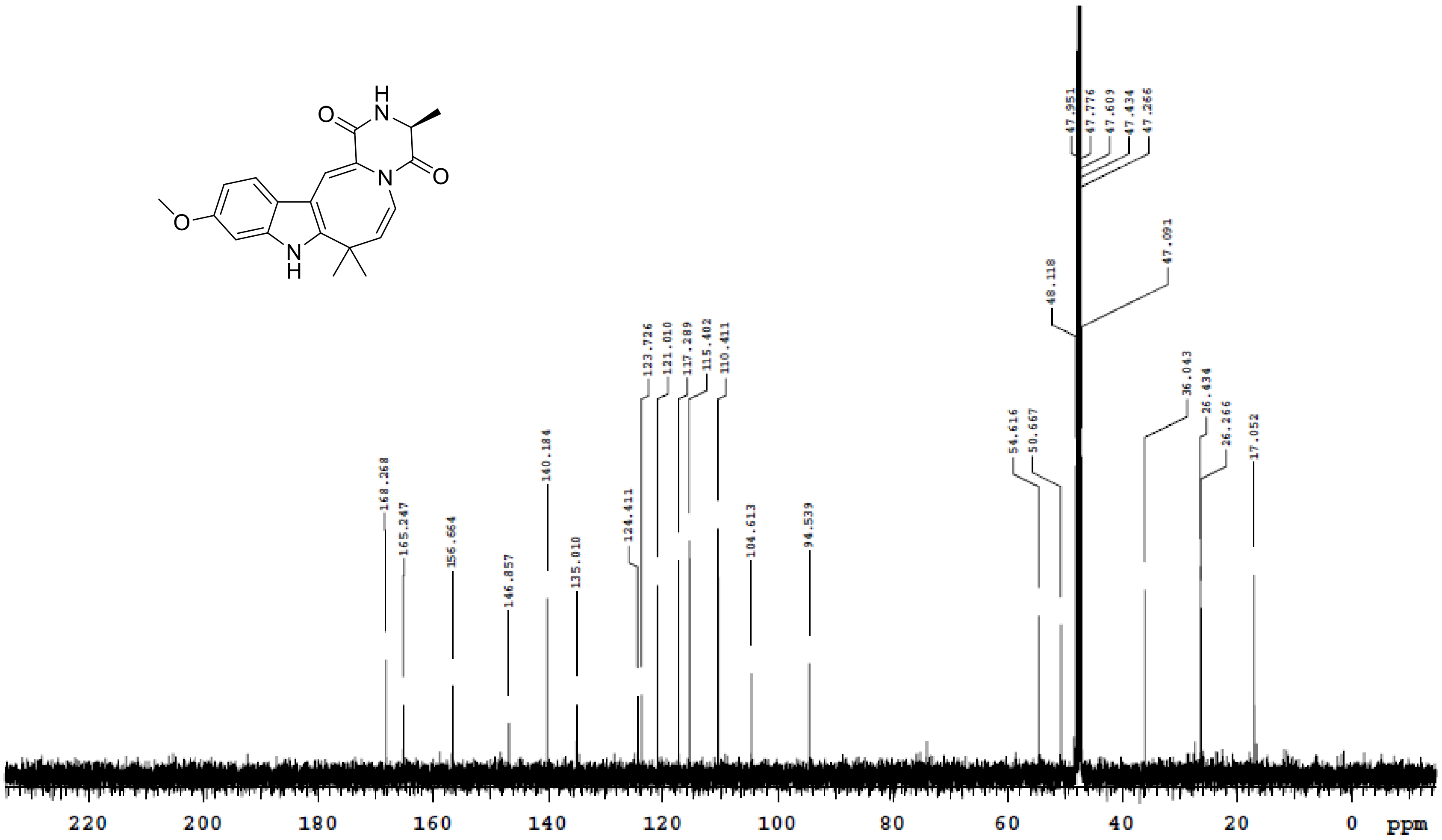

Figure S13. ^{13}C NMR spectrum of cycloechinulin (**3**).

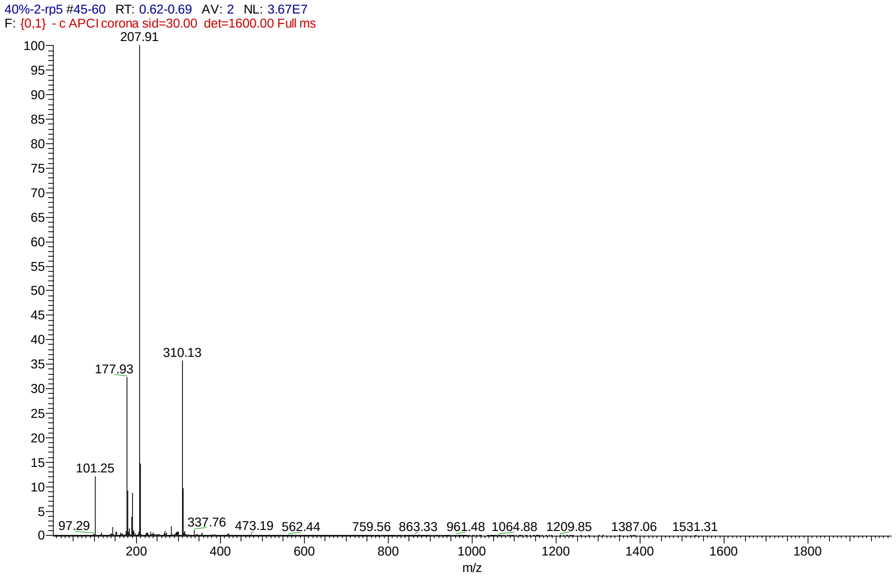

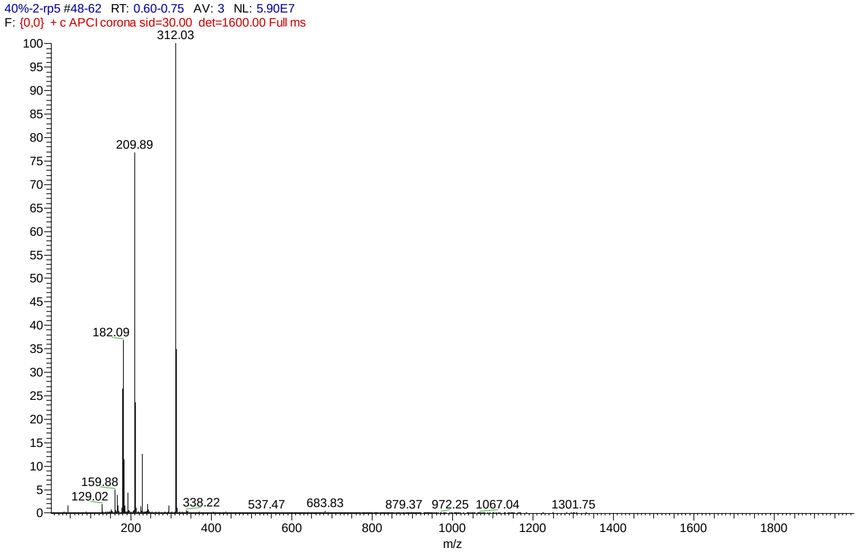

Figure S14. LRMS data of wasabidienone E (**4**).

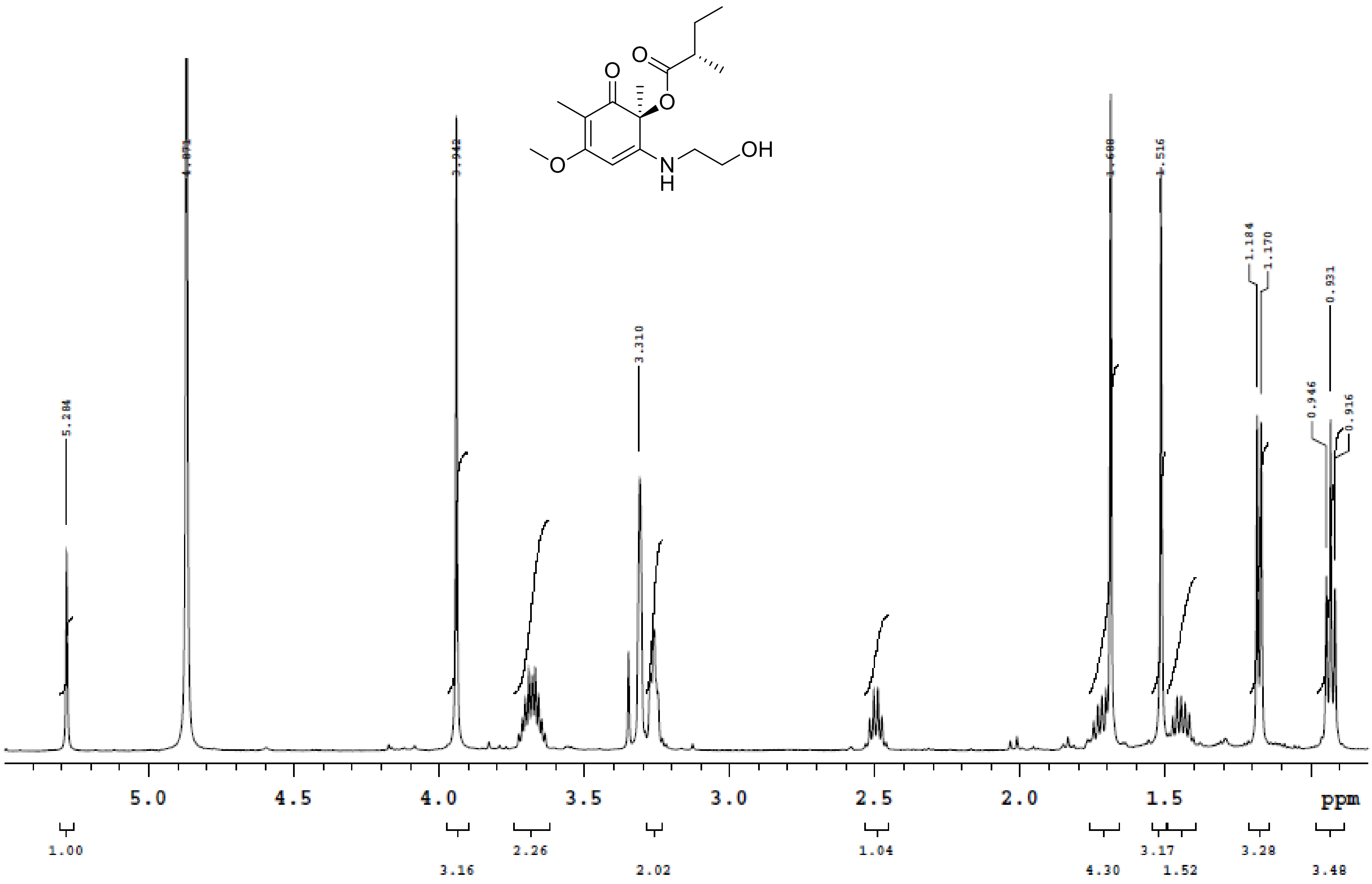

Figure S15. ^{1}H NMR spectrum of wasabidienone E (**4**).

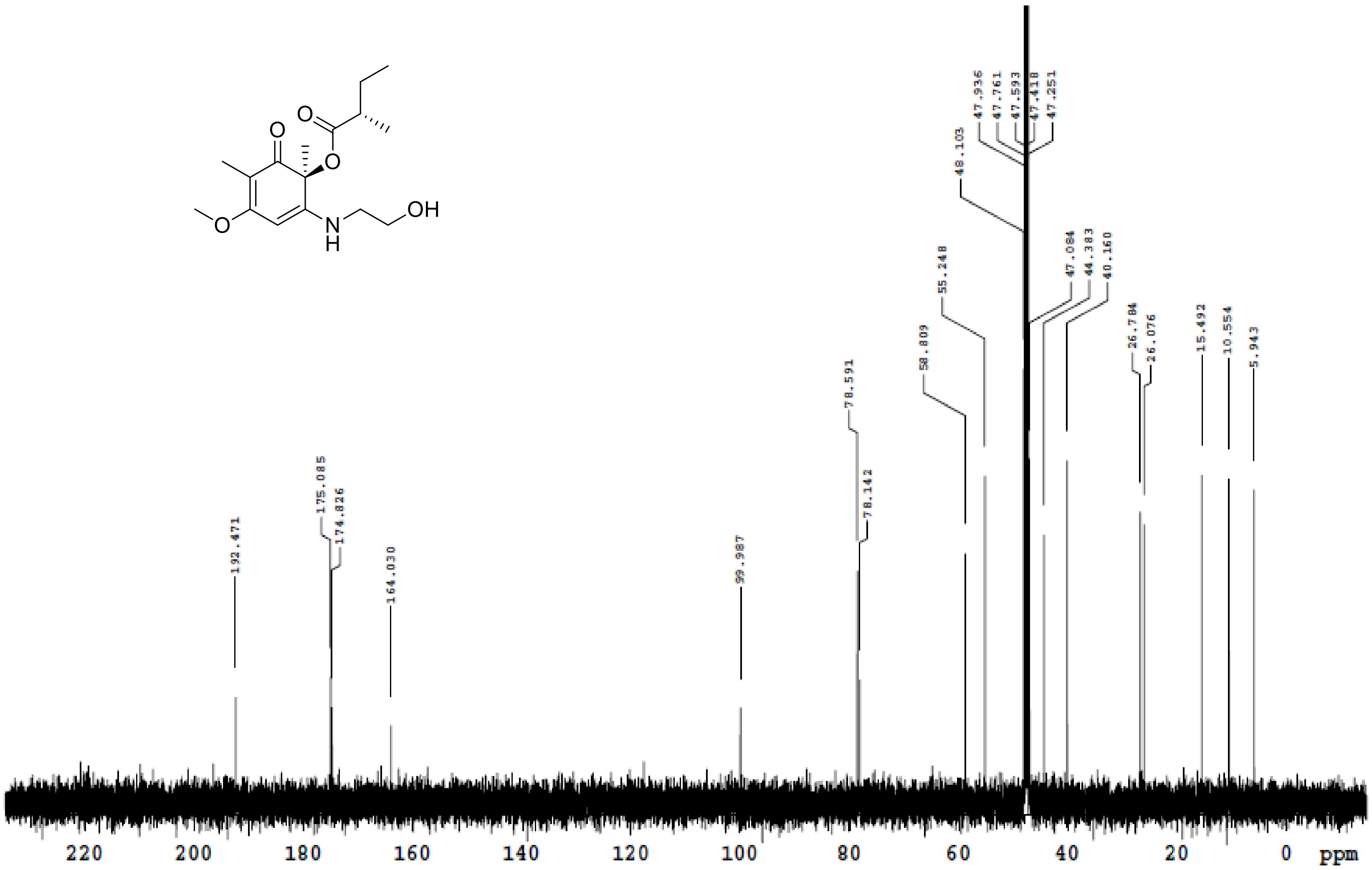

Figure S16. ^{13}C NMR spectrum of wasabidienone E (**4**).

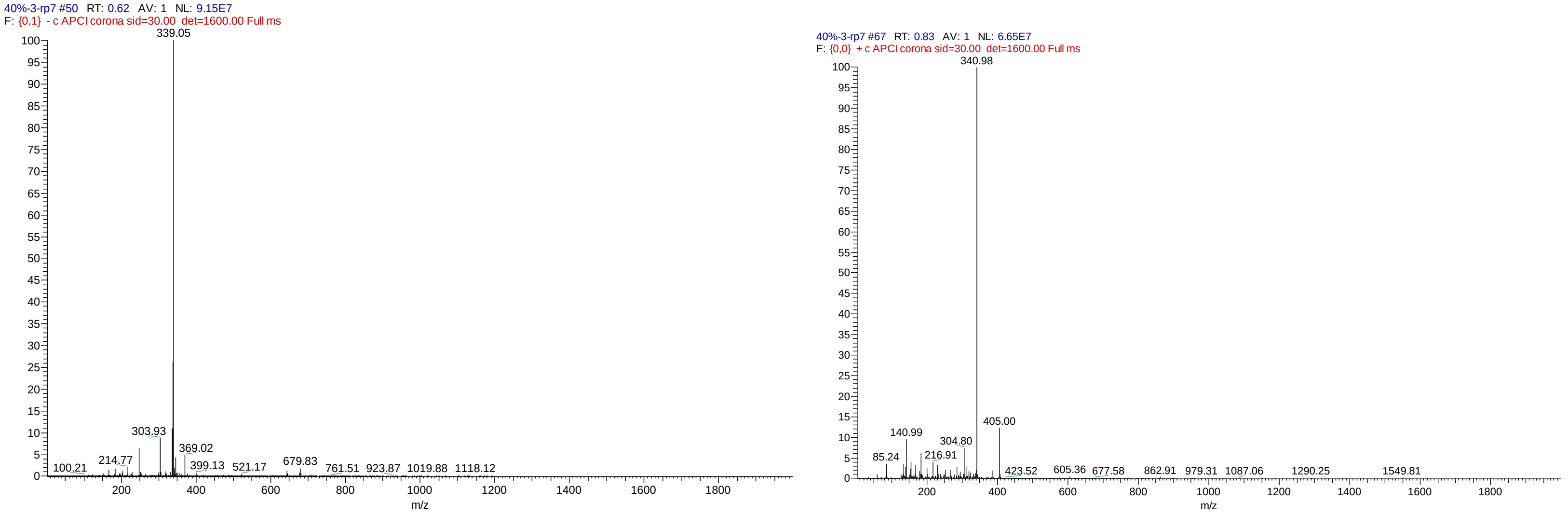

Figure S17. LRMS data of mactanamide (**5**).

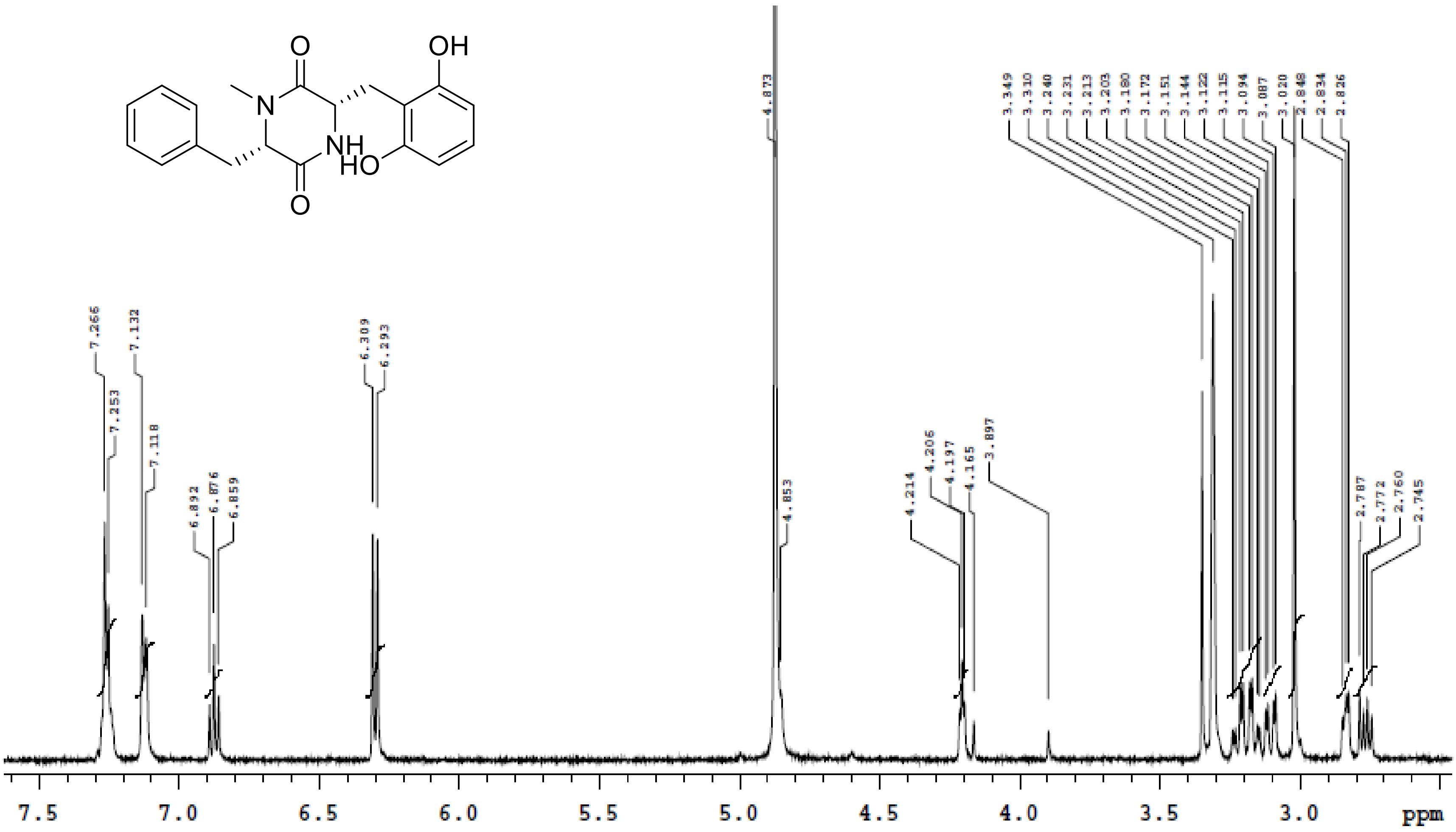

Figure S18. ^{1}H NMR spectrum of mactanamide (**5**).

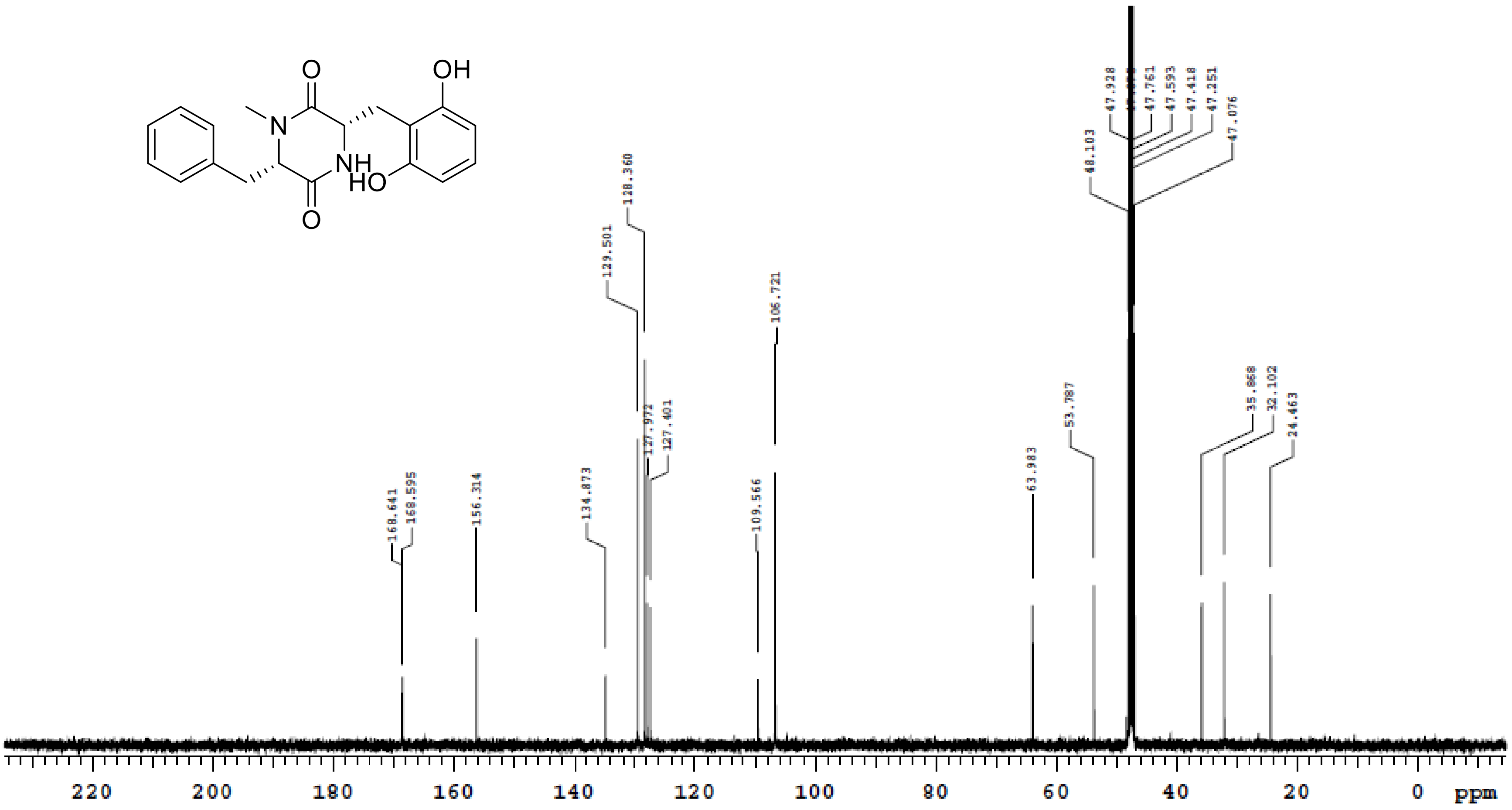

Figure S19. ^{13}C NMR spectrum of mactanamide (**5**).
